# Comprehensive plan quality assessment of simplified volumetric-modulated arc therapy for lung stereotactic body radiotherapy

**DOI:** 10.1007/s12194-025-00907-0

**Published:** 2025-05-09

**Authors:** Yuya Tatsuno, Naritoshi Mukumoto, Tomoya Ishida, Yasuyuki Shimizu, Yoshihiko Yamamoto, Satoshi Seno, Takeaki Ishihara, Daisuke Miyawaki, Ryohei Sasaki

**Affiliations:** 1 Department of Radiation Oncology, Kobe University Hospital, 7-5-2 Kusunoki-cho, Chuo-ku, Kobe, Hyogo 650-0017 Japan; 2 Center for Radiology and Oncology, Kobe University Hospital, 7-5-2 Kusunoki-cho, Chuo-ku, Kobe, Hyogo 650-0017 Japan

**Keywords:** Plan complexity, Lung SBRT, VMAT, Prescription isodose line, Plan quality

## Abstract

In lung stereotactic body radiation therapy, optimizing plan quality, including dosimetric quality and plan complexity, is paramount for mitigating adverse effects and enhancing dose delivery accuracy. This study evaluated the plan quality of dynamic conformal arc-based volumetric-modulated arc therapy (d-VMAT) as a simplified VMAT compared to conventional VMAT (c-VMAT) across various prescription isodose lines (PIL) and planning target volume (PTV) sizes. Twenty inoperable non-small cell lung cancer patients were retrospectively analyzed (PTV: 7.6–68.7 cm^3^). The prescribed dose comprised 48 Gy delivered in four fractions, encompassing 95% of the PTV, with the PIL ranging from 60 to 90% in 10% increments, using a 6X-flattening filter-free beam. The d-VMAT and c-VMAT plans were generated for each patient and PIL setting. Dose indices, including the conformity index (CI), gradient index (GI), and plan complexity, were assessed for each plan. The GI of d-VMAT closely mirrored that of c-VMAT at 60% and 70% PIL. Nevertheless, d-VMAT exhibited significantly higher GI values than c-VMAT at 80% and 90% PIL, particularly for smaller PTV sizes. Notably, d-VMAT demonstrated reduced plan complexity across all PIL compared to c-VMAT. Clinically, significant differences in CI and dose coverage between d-VMAT and c-VMAT were not observed across varying PIL settings in the range of 60–80%. The dose to the organs at risk with d-VMAT was comparable to that with c-VMAT, except at 90% PIL. In conclusion, the simplification of VMAT treatment plan using d-VMAT demonstrates superior plan quality across various PTV sizes at 60% and 70% PIL.

**Supplementary Information:**

The online version contains supplementary material available at 10.1007/s12194-025-00907-0.

## Introduction

Stereotactic body radiation therapy (SBRT) is a pivotal modality for the curative management of early-stage non-small-cell lung cancer (NSCLC) owing to its notable efficacy and minimal invasiveness [1–4]. Volumetric modulated arc therapy (VMAT) with a flattening filter-free (FFF) beam has been highlighted for its superior target conformity, coverage [5], and expedited delivery compared to coplanar dynamic conformal arc (DCA) techniques and three-dimensional conformal radiation therapy (3DCRT) [6] in SBRT treatment planning. Given the hypofractionation and high fractional dose characteristics of SBRT, precise delivery of dose distribution is imperative. At the third Physics ESTRO Workshop (2019), the actual dose distribution administered to the patient was contingent on the calculated dose distribution, robustness, and complexity of the treatment plan [7]. More complex plans typically affect the uncertainties in dose calculations due to smaller and more irregular beam apertures, larger tongue-and-groove effects, and limitations in the calculation algorithm or beam model [8, 9]. Vieillevigne et al. [9] reported that small MLC apertures and complex MLC sequences in SBRT or stereotactic radiosurgery are more sensitive to the tongue-and-groove effect and dosimetric leaf gap than those in DCA. These plans are highly sensitive to target motion during treatment, such as respiratory motion, leading to interplay effects [10–15]. Edvardsson et al. [15] conducted a comprehensive study on the interplay effects and reported that the use of FFF beams and complex treatment plans increases the impact of the interplay effects in each fraction [15]. Furthermore, highly complex plans potentially raise concerns about intra-fractional motion errors and the baseline shift or drift of the target due to the prolonged beam-on time caused by increased MU. Takao et al. investigated the frequency and amplitude of baseline shifts or drifts and reported that the occurrence of significant errors increases over time [16]. Particularly in cases such as SBRT for NSCLC, where high doses are delivered in a small number of fractions to a moving target, even minor uncertainties of the delivery dose can significantly impact the biological effect. Therefore, in lung SBRT, high treatment plan complexity should be avoided to maximize the accuracy and robustness, provided that the dose distribution is not compromised.

Several investigations have explored planning methods aimed at simplifying multi-leaf collimator (MLC) sequences. Pokhrel et al. [17] reported the clinical benefits of DCA-based VMAT (d-VMAT), which uses a DCA-based dose distribution and high-priority aperture shape control. The authors compared d-VMAT with conventional VMAT (c-VMAT) plans for ten patients undergoing single-fractional lung SBRT. Their findings revealed favorable dosimetric quality and reduced delivery time attributable to simpler MLC modulation and diminished total monitor unit (MU) with d-VMAT. Bokrantz et al. [18] demonstrated the effectiveness of a segment-weight-optimized DCA coupled with an inverse planning technique in lung SBRT using RayStation (RaySearch Laboratories, Stockholm, Sweden). Similarly, Darréon et al*.* [19] illustrated that VMAT with leaf speed limitations yields decreased plan complexity without compromising dosimetric quality compared to c-VMAT. However, a conclusive statement that simplified VMAT techniques offer equivalent dosimetric quality across diverse clinical scenarios cannot be ensured because of their significantly reduced complexity compared to c-VMAT. Hence, further comprehensive investigation is warranted in clinically relevant scenarios in which the simplified VMAT is advantageous.

In lung SBRT, using a lower prescription isodose line (PIL) results in high gross tumor volume (GTV) doses and steeper dose gradients. Oku et al. [20] reported that PIL correlates with the mean target and lung doses in the multiple DCA techniques. Chan et al. [21] investigated the optimal PIL in the inverse optimization of VMAT for SBRT in NSCLC. Their findings indicated that PIL influences the dosimetric quality of the lung SBRT plan. Consequently, the PIL is also considered to influence dosimetric quality in simplified VMAT. Furthermore, the target size may also affect the quality of the dose distribution.

Hence, this study comprehensively evaluated plan quality, encompassing dosimetric quality and plan complexity of d-VMAT as a simplified VMAT across varying PILs and planning target volume (PTV) size compared to c-VMAT. Furthermore, it explored the clinical situation under which the simplification of VMAT treatment planning benefits lung cancer SBRT.

## Materials and methods

### Patient selection and target definition

Twenty patients with early-stage NSCLC who underwent lung SBRT were enrolled in this study. Patient characteristics are summarized in Table 1. This study was approved by the Institutional Review Board (IRB No. B230237) at the Kobe University Hospital. The internal target volume (ITV) sizes ranged from 1.5 to 30.6 cm^3^, while the PTV sizes ranged from 7.6 to 68.7 cm^3^. All patients were immobilized using the Vaclok immobilization system (Engineering System Co., Ltd., Nagano, Japan) and underwent four-dimensional computed tomography (4DCT) using a real-time positioning management system (RPM, Varian Medical Systems, Palo Alto, CA, USA). Additionally, free-breathing planning 3DCT images were acquired using a CT scanner (Aquilion large bore; Canon Medical Systems, Otawara, Japan) with a resolution of 512 × 512 pixels for slice thickness of 1 mm. The ITV was defined based on the 4DCT on 3DCT. PTV was defined as ITV plus isotropic 5 mm 3D margin. The lungs, spinal cord, ribs, heart, and trachea were contoured as organs at risk (OAR). The planning organ at risk volume (PRV) was generated by adding a 3 mm isotropic margin to the spinal cord.

**Table 1:** Characteristics of the 20 patients including in this study

Patient no	ITV volume (cm^3^)	PTV volume (cm^3^)	Tumor location
1	7.1	24.2	Right upper lobe
2	2.4	10.5	Left upper lobe
3	17.8	44.5	Right lower lobe
4	7.5	23.5	Right upper lobe
5	30.6	68.7	Left upper lobe
6	14.8	40.4	Right upper lobe
7	3.5	16.1	Right lower lobe
8	12	32.7	Right upper lobe
9	3.4	15.3	Left upper lobe
10	10.2	30.2	Right upper lobe
11	24.8	55.9	Right upper lobe
12	4.4	16.7	Right upper lobe
13	34.6	68.7	Left lower lobe
14	8.8	25.7	Right upper lobe
15	6.0	19.6	Left lower lobe
16	3.9	14.1	Left upper lobe
17	4.3	15.4	Right upper lobe
18	9.8	27.1	Right upper lobe
19	1.5	7.6	Right upper lobe
20	5.7	19.1	Right upper lobe

### Treatment planning

Varying PIL treatment plans were generated using a treatment planning system (Eclipse ver.15.6, Varian Medical Systems, Palo Alto, CA, USA) for TrueBeam STx with HD120 MLC, using a 6X-FFF beam. The prescribed dose of 48 Gy was delivered in four fractions to cover 95% of the PTV. Four plans with PILs set at 60%, 70%, 80%, and 90% were generated using two treatment planning techniques for each patient: d-VMAT [17] as a simplified plan and c-VMAT as a high-complexity plan. Therefore, eight plans were generated for each patient.

#### Treatment planning for c-VMAT

The c-VMAT treatment plan (181°–30° or 330°–179° partial coplanar two arc, collimator angles of 30° and 330°) with aperture shape controller (ASC) off for varying PIL was optimized. The dose-volume histogram (DVH) parameters for the GTV were adjusted to achieve varying PIL during the VMAT optimization. The dose constraints for the OAR, ring structure, and normal tissue objectives (NTO) remained fixed during all inverse optimizations for each patient to facilitate the comparison of treatment planning methods. The final dose distribution was calculated with an Anisotropic Analytical Algorithm (AAA) using a 1.25 mm calculation resolution and heterogeneity correction.

#### Treatment planning for d-VMAT

**Fig. 1:**
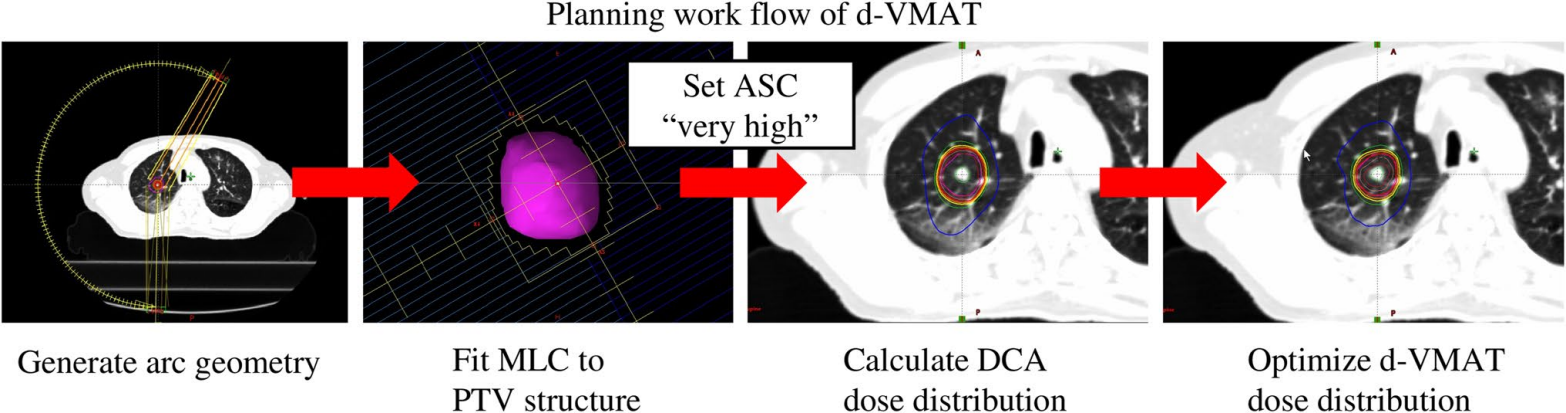
Illustration of d-VMAT treatment planning workflow. The calculated DCA dose was used as the intermediate dose for VMAT optimization

The d-VMAT treatment plan for varying PIL was reoptimized. The treatment planning workflow for d-VMAT is shown in Fig. 1. First, the DCA plan (181°–30° or 330°–179° partial coplanar two arc, collimator angles of 30° and 330°) was generated using the arc geometry tool. MLCs were fitted to the PTV with − 2, 0, 2, and 5 mm MLC margins for the 60%, 70%, 80%, and 90% PIL plans, respectively. Subsequently, a very high priority in the ASC was selected using the Photon Optimizer (PO) and the 3D volume dose distribution was calculated using the AAA ver.15.6.06 with heterogeneity correction. After creating the dose distribution for DCA, normalization was set to off, and the VMAT optimization was restarted. The option “use the current plan as an intermediate dose for optimization” was selected, and the process returned to MR Level 1 to continue the VMAT optimization calculation. The final dose distribution was calculated using grid sizes of the same dose calculation, employing the same algorithms as in c-VMAT.

### Plan quality evaluation and statistical analysis

The d-VMAT and c-VMAT plans for varying PIL were compared using several dose indices, including the dose to 98% of the PTV (*D*_98%_), dose to 2% of the PTV (*D*_2%_), minimum dose to the ITV (*D*_100%_), maximum dose to the PRV of the spinal cord, percentage of bilateral lungs receiving a dose equal to or exceeding 20 Gy (Lung *V*_20Gy_), and percentage of bilateral lungs receiving a dose equal to or exceeding 5 Gy (Lung *V*_5Gy_). Dose constraints for the ribs were excluded due to the overlap between the PTV and the ribs in some cases. Additionally, in this study, all registered cases had peripheral lung cancer. As the doses to the heart, esophagus, and great vessels were sufficiently low, they were excluded from the evaluation. Target conformity was assessed using the Paddick conformity index (CI) [22]. The CI is defined by the following equation:

$$\text{CI}= \frac{{\text{TV}}_{\text{PIV}}^{2}}{\text{TV} \times \text{PIV}},$$

where TV_PIV_ denotes the target volume covered by the prescribed isodose, TV is the PTV volume, and PIV is the volume covered by the prescribed isodose. The dose gradient was evaluated using a gradient index (GI) [23] defined as follows:

$$\text{GI}= \frac{{V}_{50\%}}{\text{PIV}},$$

where *V*_50%_ corresponds to the volume covered by a dose that is equal to or greater than 50% of the prescribed dose. The dose gradient for the d-VMAT plan was assessed and compared with that for c-VMAT. Moreover, we assessed the relationship between the PTV size and GI for each plan and PIL. Plan complexity was assessed using the number of total monitor unit (MU) and the modulation complexity score for VMAT (MCSv) [24]. We calculated MCSv using the Eclipse Scripting API (ESAPI). Additionally, we measured the beam-on time for d-VMAT plan and c-VMAT plan for 70% PIL. The Wilcoxon signed-rank test (p < 0.05, considered statistically significant) was employed to assess the statistical significance of the target and OAR dose indices, CI, GI, and plan complexity parameters (MU and MCSv).

## Results

### Dosimetric quality

**Fig. 2:**
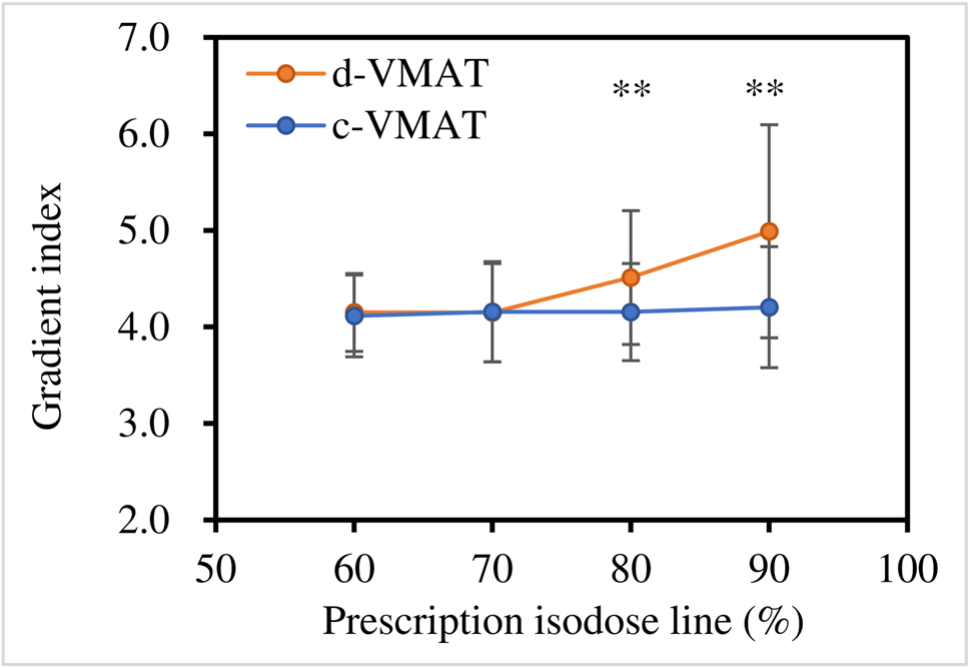
Comparison of gradient index between d-VMAT and c-VMAT at varying PIL. *PIL* prescription isodose line

**Fig. 3:**
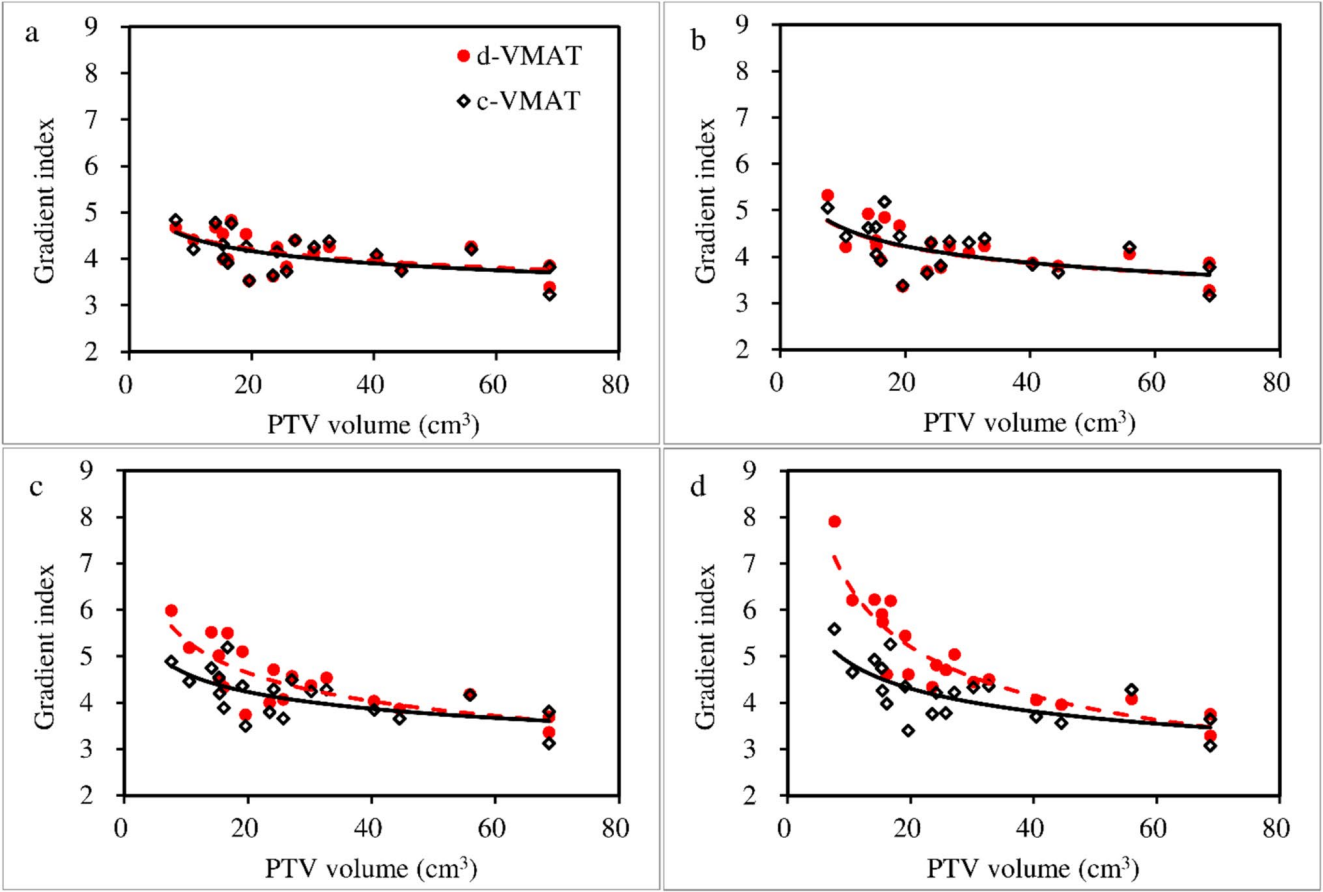
This figure illustrates the relationship between PTV size and gradient index for d-VMAT and c-VMAT at 60% (**a**), 70% (**b**), 80% (**c**), and 90% (**d**) PIL plans. PIL: Prescription isodose line

**Fig. 4:**
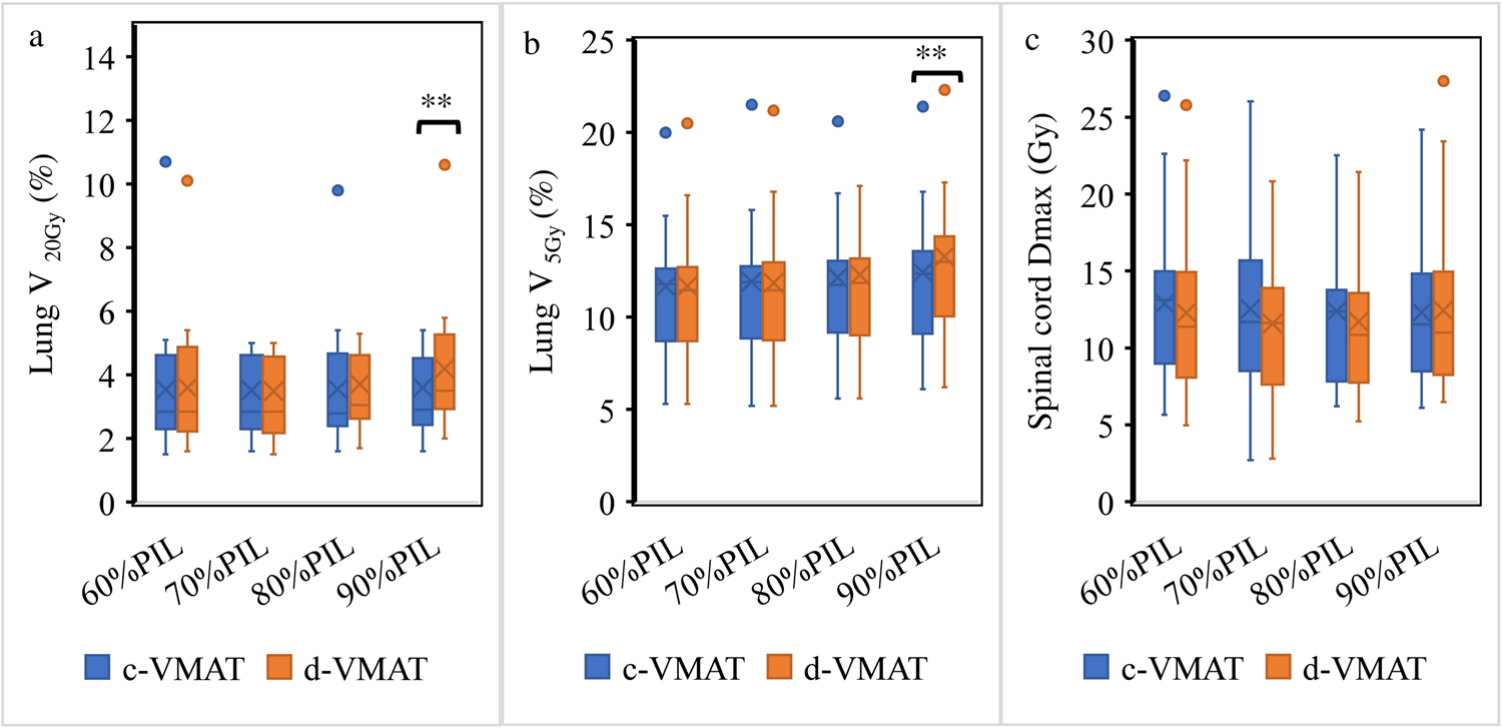
*V*_20Gy_ and *V*_5Gy_ for bilateral lungs (**a**), (**b**) and *D*_max_ for spinal cord (**c**)

**Fig. 5:**
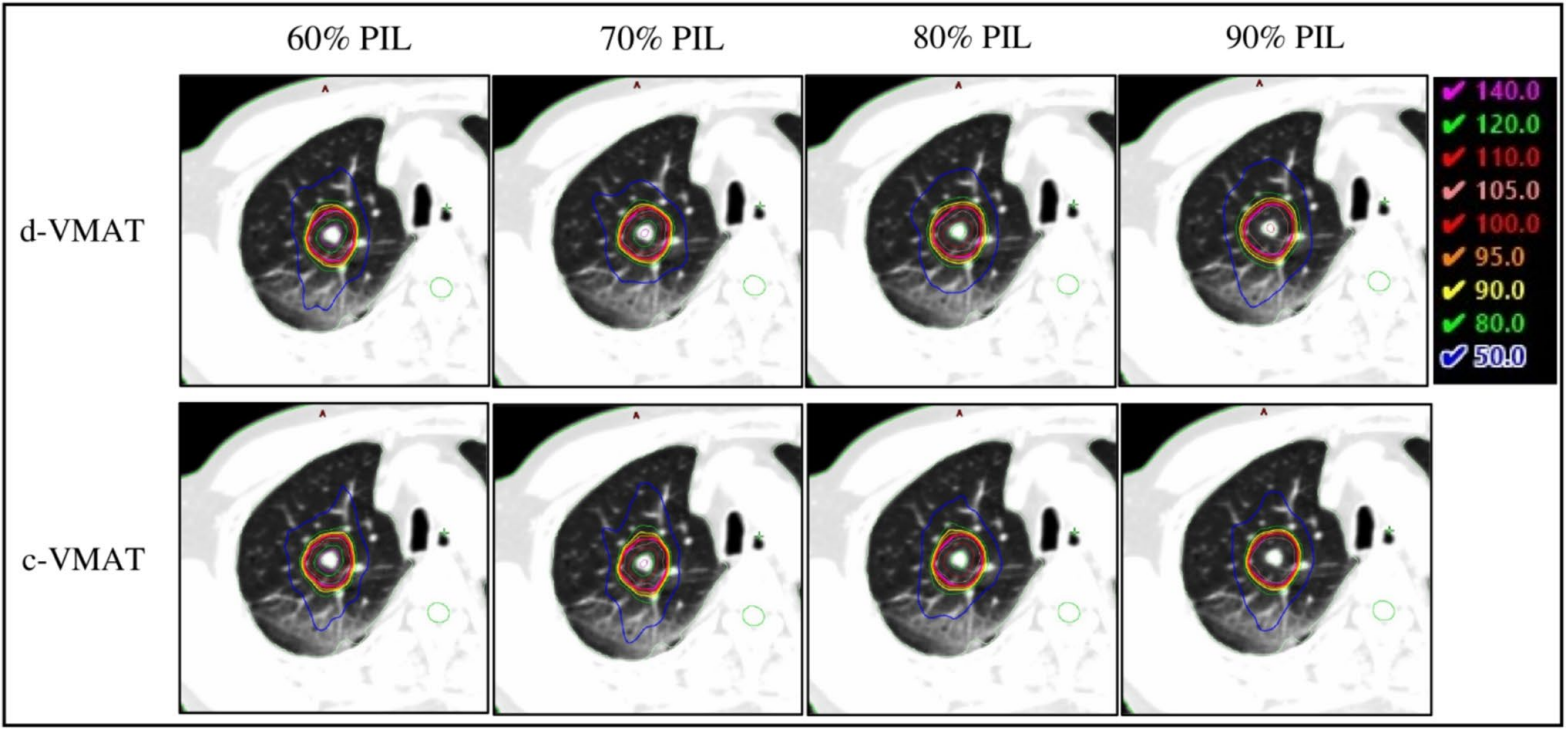
Dose distribution of patient #2 with a small PTV size for varying prescription isodose levels. The upper panel displays the dose distribution of d-VMAT. The lower panel shows the dose distribution of c-VMAT

No significant differences were noted in the gradient index between d-VMAT and c-VMAT at 60 and 70% PIL, indicating comparable dose gradients outside the target when d-VMAT was used at these PILs (Fig. 2). However, GI of d-VMAT was significantly higher than that of c-VMAT at 80% (4.51 ± 0.69 versus 4.16 ± 0.50, *p* < 0.01) and 90% PIL (4.99 ± 1.10 versus 4.21 ± 0.63, *p* < 0.01). The GI of d-VMAT was comparable to that of c-VMAT at 60 and 70% PIL, irrespective of PTV size (Fig. 3). Nevertheless, the GI of d-VMAT tended to be higher than that of c-VMAT at 80 and 90% PIL, particularly for small PTVs. This implies that d-VMAT at 80 and 90% PIL for a small PTV reduced dosimetric quality compared to c-VMAT. The dose metrics for the targets of c-VMAT and d-VMAT for various PILs are summarized in Table 2. The mean ± 1 SD of the conformity index for c-VMAT and d-VMAT at 90% PIL were 0.91 ± 0.02 and 0.88 ± 0.02 (*p* = 0.017), respectively. The target conformity of d-VMAT was significantly lower than that of c-VMAT at 90% PIL. Significant differences were observed in PTV *D*_98%_, *D*_2%_ and ITV *D*_100%_. However, these were deemed to have a slight effect on clinical practice. No significant differences in OAR dose indices between c-VMAT and d-VMAT were observed at 60%, 70% and 80% PIL (Fig. 4). However, significant differences were observed in lung *V*_20Gy_ and *V*_5Gy_ at 90% PIL (*p* < 0.01). Figure 5 displays an example of the dose distribution of lung SBRT for Patient 2, with small PTV sizes at 60%, 70%, 80%, and 90% PIL (the upper and lower panels show the d-VMAT and c-VMAT plans, respectively). Moreover, d-VMAT achieved comparable target conformity and a tight 50% isodose line compared with c-VMAT at 60 and 70% PIL. However, the intermediate-dose spillage of d-VMAT was more expanded than that of c-VMAT at 80 and 90% PIL.

**Table 2:** Dose metrics for target of c-VMAT and d-VMAT

	PTV		PTV		ITV		Conformity index	
	D98%		D2%		D100%			
PIL	c-VMAT	d-VMAT	c-VMAT	d-VMAT	c-VMAT	d-VMAT	c-VMAT	d-VMAT
60%PIL	**46.92 ± 0.20**	**46.59 ± 0.49**	**78.22 ± 1.70**	**77.35 ± 2.18**	**53.22 ± 0.67**	**53.96 ± 1.16**	0.90 ± 0.02	0.90 ± 0.02
70% PIL	47.19 ± 0.15	47.15 ± 0.20	**66.68 ± 1.64**	**66.13 ± 1.76**	**51.22 ± 0.48**	**51.56 ± 0.59**	0.91 ± 0.01	0.92 ± 0.02
80% PIL	47.18 ± 0.22	47.28 ± 0.14	59.80 ± 0.81	59.62 ± 0.96	51.09 ± 0.96	51.19 ± 0.49	0.91 ± 0.02	0.91 ± 0.02
90% PIL	47.20 ± 0.08	47.19 ± 0.09	**51.55 ± 0.37**	**51.91 ± 0.51**	**49.61 ± 1.11**	**50.35 ± 1.38**	**0.91 ± 0.02**	**0.88 ± 0.03**

The number in bold represent values that reached statistical significance (p <0.05)

### Plan complexity

**Fig. 6:**
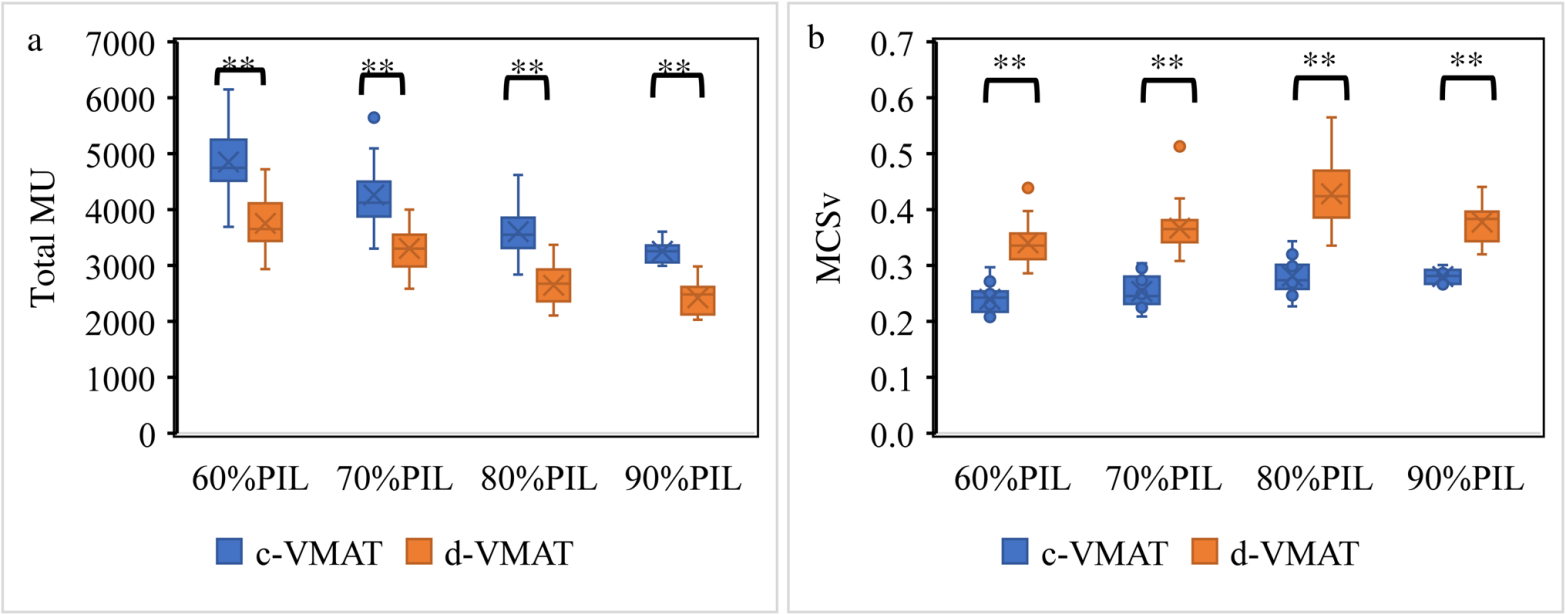
The left panel illustrates the total number of MU for c-VMAT and d-VMAT for varying PIL (**a**). The right panel displays the MCSv for c-VMAT and d-VMAT with varying PIL (**b**). MCSv: Modulation complexity score for VMAT

**Fig. 7:**
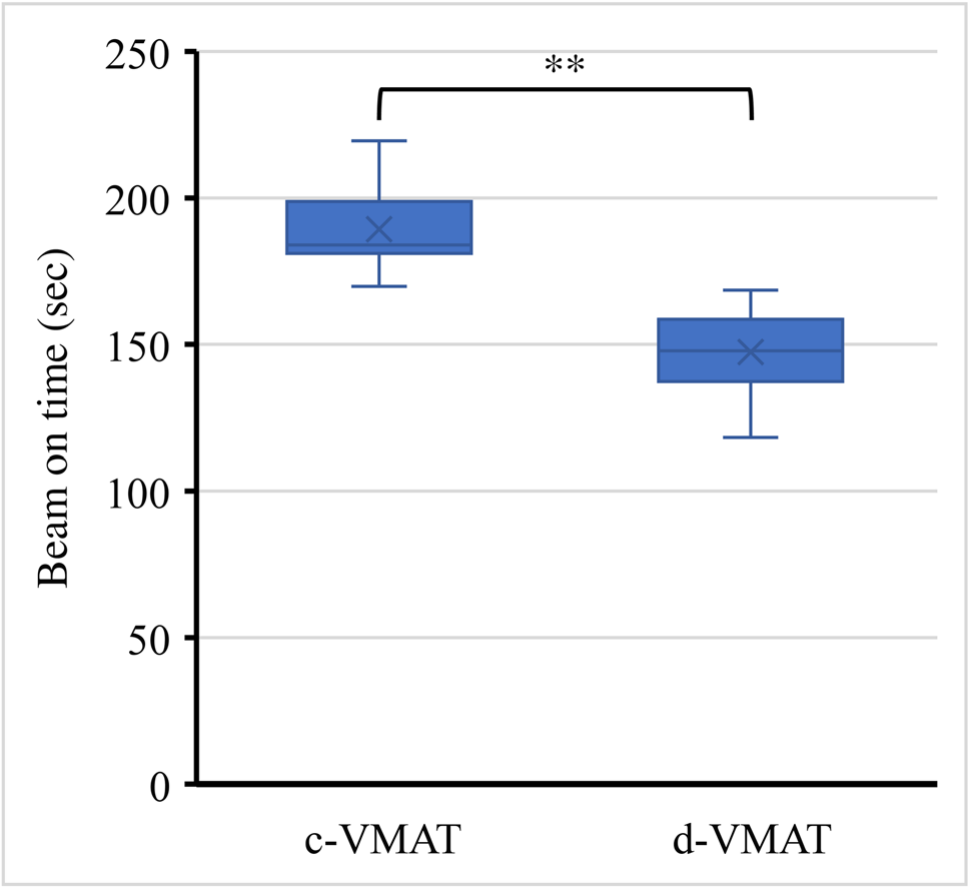
Beam-on time for d-VMAT and c-VMAT at 70% PIL

Plan complexity was evaluated using the total number of MU and the MCSv. The d-VMAT plan significantly reduced the total number of MU compared to the c-VMAT plan for all PIL (Fig. 6a). The total number of MUs of the c-VMAT and d-VMAT plans at 60%, 70%, 80%, and 90% PIL was 4853.1 ± 548.7 and 3755.1 ± 417.0, 4408.7 ± 554.8 and 3340.3 ± 341.2, 3639.6 ± 220.2 and 2716.0 ± 289.8, and 3247.8 ± 202.3 and 2438.9 ± 289.2, respectively. The d-VMAT plan also exhibited a significantly higher MCSv than the c-VMAT plans (Fig. 6b), indicating that the d-VMAT plans had lower MLC modulation complexity than the c-VMAT plans for all PIL. Figure 7 shows the beam-on-time for c-VMAT and d-VMAT at 70% PIL. The beam-on time of the c-VMAT and d-VMAT plans at 70% PIL were 189.3 ± 13.9 and 147.4 ± 13.9 s. Hence, d-VMAT achieved dosimetric quality comparable to c-VMAT at 60 and 70% PIL while reducing MU, beam-on-time, and plan complexity.

## Discussion

The plan quality of the simplified VMAT for two partial coplanar-arc lung SBRT with varying PIL was assessed and compared to that of conventional VMAT to explore the conditions under which simplification of the VMAT treatment plan is advantageous. In particular, we evaluated the plan quality for d-VMAT as a simplified plan and c-VMAT as a high-complexity plan, focusing on dose distribution and plan complexity. The d-VMAT plan showed comparable target conformity, target coverage, and dose gradient to c-VMAT at 60% and 70% PIL. However, the d-VMAT plan displayed a higher GI at 80% and 90% PIL, particularly for small PTV sizes (Fig. 3). For example, Ding et al. [25] suggested that an optimal PIL of approximately 60–70% is suitable for lung SBRT using 3DCRT. Oku et al. [17, 20] reported that a PIL of 60% is superior to the reference 80% PIL in term of lower lung dose. Furthermore, at lower PILs, a steep dose gradient was achieved regardless of complexity of the treatment plan (Figure S1). These facts suggest that, at lower PILs, where a steep dose gradient is achieved with DCA, a complex MLC sequence is not necessary to achieve a steep dose gradient and high target conformity. Moreover, at a 90% PIL, a uniform dose distribution within the target was required. In c-VMAT, a complex MLC sequence that traverses the target enables the achievement of a uniform dose distribution within the target while preserving the steep dose gradient. In d-VMAT, the limited traversal of the MLC sequence across the target requires the expansion of the irradiation field to achieve a uniform dose distribution within the target (Figure S2). Consequently, the GI of d-VMAT deteriorated at 90% PIL. In addition, at a 90% PIL, the CI slightly deteriorated for d-VMAT. Because d-VMAT was optimized based on the dose distribution from DCA, significant adjustments to the dose distribution were required during optimization at the 90% PIL, where the CI deteriorated in the base plan using the DCA technique. It is hypothesized that the complexity of the MLC in d-VMAT is insufficient to accommodate these adjustments, leading to slight degradation of the CI. As shown in Fig. 6, MCSv for d-VMAT deteriorates at 90% PIL compared to 80% PIL. This result suggested that a more complex MLC sequence is required at 90% PIL. In terms of dosimetric quality, d-VMAT, a simplified form of VMAT, can provide comparable quality to c-VMAT at 60% and 70% PIL. However, in the 80% and 90% PIL ranges, particularly for small PTV sizes, simplifying the MLC sequence can degrade dosimetric quality. In such clinical situations, simplification of VMAT plans should be approached cautiously when compared to the dose distribution of c-VMAT.

Furthermore, d-VMAT showed a higher MCSv than c-VMAT across all PILs, indicating a reduction in plan complexity. Edvardsson et al. [15] reported that the interplay effect between respiratory-induced target motion and dynamic MLC sequences was more pronounced for highly modulated plans and FFF beam plans. In their study, a maximum difference of approximately 16% in D_98%_ and D_2%_ was observed for complex treatment plans, depending on the breathing phase at the start of irradiation. Similarly, Ge et al. [26] found that highly modulated VMAT SBRT plans with FFF beams provided limited benefits on dosimetric quality but compromised delivery accuracy and increased motion-induced interplay effects. Gauer et al. [27] highlighted that abnormal respiratory motion combined with a highly modulated plan enhanced the interplay effect. These reports suggest that complex treatment plans may increase the interplay effect in SBRT for lung cancer with respiratory motion. Therefore, in this case, a simplified plan similar to d-VMAT could potentially be an alternative to complex VMAT to reduce plan complexity. In a previous study, MCSv [23] was used to assess plan complexity. Sande et al. [28] reported a moderate correlation between MCSv and the mean measured motion-induced dose deviation for lung SBRT treatment. Therefore, we used MCSv as a robustness metric for the interplay effect in lung SBRT plans. Additionally, d-VMAT reduced the total number of MU by approximately 25% compared with c-VMAT. Consequently, the total beam-on-time was reduced by approximately 25% in the 70% PIL case, which is consistent with the results reported by Pokhrel et al. [17]. This reduction benefits the reduction of intra-fractional motion errors and, in the case of breath-hold irradiation, alleviates the burden on the patients. The complexity of the treatment plan is viewed as an assessment of its robustness against these uncertainties in dose delivery. Therefore, if equivalent dose distributions can be achieved, simplified treatment plans can be considered advantageous from the dose delivery perspective.

However, our study had limitations. First, we did not directly measure the interplay effect in d-VMAT and c-VMAT. Multiple studies have reported that complex MLC movements enhance interplay effects [15, 26, 27]. Here, d-VMAT demonstrated a significant reduction in the complexity of the MLC sequence; however, a comprehensive investigation of the interplay effect in d-VMAT is required. Second, this study did not validate the Acuros XB algorithm, which is an additional limitation. Nevertheless, this study utilized the same dose calculation algorithm for both d-VMAT and c-VMAT, ensuring a fair comparison. Third, our study only included patients with peripheral tumors, warranting further investigation of the central lung region, which requires a more complex dose distribution.

Essentially, d-VMAT is an effective simplified alternative to c-VMAT for lung SBRT at lower PILs (60% and 70%), offering comparable dosimetric quality with fewer MUs and lower complexities.

## Conclusion

We investigated and compared the plan quality of simplified VMAT with varying PIL to that of conventional VMAT in SBRT for early-stage NSCLC. The d-VMAT plan at 60% and 70% PIL achieved comparable dosimetric indices, including target coverage, dose gradient, and OAR dose, while fewer MU and exhibiting fewer MLC modulations. Simplified VMAT may offer a higher plan quality via dosimetric quality and plan complexity at lower PIL levels. However, carefully considering the PIL and PTV sizes is essential to effectively optimize the plan quality for lung SBRT.

## Supplementary Information

Below is the link to the electronic supplementary material.

Supplementary file1 (DOCX 437 KB)

## Data Availability

The data in this study are stored in our laboratory and will be shared upon request by the corresponding author.
