# Supplementary material for: Comprehensive plan quality assessment of simplified volumetric-modulated arc therapy for lung stereotactic body radiotherapy
Source: Radiol Phys Technol. 2025 May 9;18(2):547–55. doi: 10.1007/s12194-025-00907-0 (PMC12103334; doi:10.1007/s12194-025-00907-0)
Supplement: Supplementary file 1 — Supplementary file1 (DOCX 437 KB) [file 12194_2025_907_MOESM1_ESM.docx]

Supplementary file 1

A lung cancer stereotactic body radiation therapy (SBRT) plan was created for an inhomogeneous phantom (The PTV sizes is 24.5 cm^3^) with a total dose of 48 Gy delivered in 4 fractions (12 Gy per fraction) using a 6X-flattening filter- free beam. For treatment plans with varying complexities—DCA, d-VMAT, and c-VMAT—plans were generated at 10% increments from 60% to 90% PIL, and the GI was evaluated. In the 60–80% PIL range, in which the dose distribution within the target was inhomogeneous, a comparable dose gradient was achieved regardless of the plan complexity (Figure S1). These results suggest that when delivering an inhomogeneous dose within the target, a complex MLC sequence is not required to achieve a steep dose gradient outside the target. In contrast, at 90% PIL, where a relatively homogeneous dose distribution within the target was required, the GI worsened significantly as the treatment plan was simplified (Figure S1). These results suggest that achieving a steep dose gradient outside the target, while maintaining a uniform dose distribution within the target, requires a complex MLC sequence across the target (Figure S2).


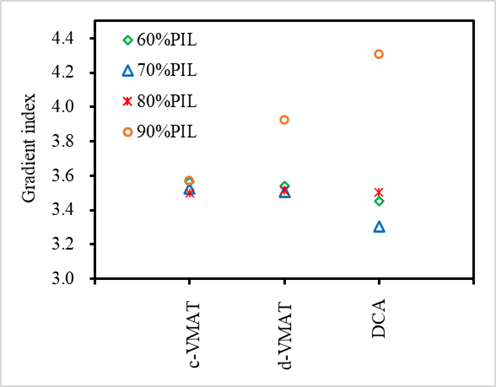


Figure S1. GI according to the SBRT plan with varying complexity


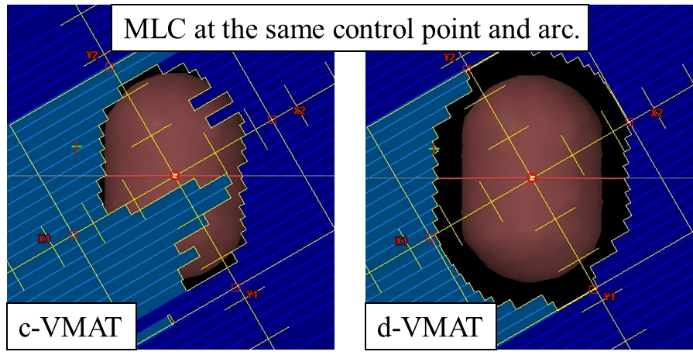


Figure S2. Comparison between the MLC aperture shape at the same control point at 90% PIL
